# Transition Pathways Toward the Prudent Use of Antimicrobials: The Case of Free-Range Broiler Farmers in France

**DOI:** 10.3389/fvets.2020.548483

**Published:** 2020-10-07

**Authors:** Cécile J. M. Adam, Nicolas Fortané, Christian Ducrot, Mathilde C. Paul

**Affiliations:** ^1^IHAP, ENVT, INRAE, Université de Toulouse, Toulouse, France; ^2^UMR EPIA, INRAE, VetAgroSup, Saint-Genès-Champanelle, France; ^3^UMR IRISSO, CNRS, INRAE, Université Paris Dauphine, PSL, Paris, France; ^4^London School of Hygiene and Tropical Medicine (LSHTM), London, United Kingdom; ^5^ASTRE, Univ Montpellier, CIRAD, INRAE, Montpellier, France

**Keywords:** antibiotics, poultry, behavioral change, farming practices, animal health, veterinary medicine, qualitative approach, social sciences

## Abstract

Reducing antimicrobial use (AMU) on farms is key for controlling the rise of resistant bacteria that have the potential capacity to infect humans *via* direct animal contact or *via* the food chain or the environment. To reduce AMU, antimicrobials must be used in a prudent and rational manner. Extensive efforts have been made recently to identify the cognitive and behavioral barriers to the appropriate use of antimicrobials by various livestock sector stakeholders. However, most studies carried out thus far have only partly captured the dynamic and systemic dimension of the processes involved in changes of practices related to AMU on farms. To shed light on the transition pathways implemented to reduce AMU, a qualitative study was conducted in France based on 28 semi-structured interviews with farmers, technicians and veterinarians from the free-range broiler production sector. Based on the thematic analysis of verbatims, we identified technical improvements which are key contributors to reduced AMU. We also highlighted some gaps in knowledge regarding AMU and antimicrobial resistance. We found that, rather than individual motivations alone, the extent to which farmers are embedded in collective organizations is decisive for changes in practices, and downstream operators (distributors and slaughterers) play a key role in the beginning of AMU transition pathways. As a result, we show that change in AMU requires a global rethinking of the overall socio-technical system rather than modifications of a single element in a farming system. Our results also highlight that transition pathways toward reduced AMU cannot just rely on trigger events, but also involves medium or long-term processes, with actors' experiences and practices being modified on an incremental basis over time. Our study sheds light on the need for multi and trans-disciplinary research involving the social sciences to analyze interactions between stakeholders and the collective actions implemented to tackle the challenge of AMU reduction.

## Introduction

Antimicrobial use (AMU) on farms is contributing to the rise of resistant bacteria that have the potential capacity to infect humans *via* direct animal contact or *via* the food chain or the environment, representing a major threat to human health (1). Antimicrobial resistance (AMR) is an increasing problem and has limited the effective lifespan of newly developed antimicrobial compounds to only 10–20 years (2). The recent growing awareness of AMR as a global public health threat has catalyzed the implementation of regulatory and voluntary public policies aiming to curb AMU and ensure antimicrobial stewardship to slow down the erosion of susceptibility or even decrease resistance of bacteria to antimicrobials. The main objective of numerous action plans implemented recently has been to reduce AMU. It should be noted that these plans are part of a longer-term dynamic involving the development of guidelines and incentives to favor the rationale or prudent use and prescription of antibiotics that date back to the late 1960s (3). Efforts to reduce AMU in the food animal production sector are complicated, however, by the fact that in addition to treating diseases, farmers use antimicrobials to keep their herds healthy and highly productive while ensuring animal welfare and food safety objectives. Antibiotics have been used as growth promoters in livestock farming since the late 1940s, and although this kind of use has been banned in Europe since 2006, it is still a major issue worldwide (4, 5). Managing tradeoffs between massive restriction of AMU and maintenance of current and potentially non-sustainable consumption levels poses a huge challenge to public policies (1).

Significant change in AMU on farms has been observed in recent years following public policies implemented in various countries (6–10). In France, an important decrease in AMU was observed after the first EcoAntibio plan was launched in 2011. While the plan's objectives have been achieved, efforts are continuing, in particular for intensive breeding productions (11), and a second plan is now underway. Extensive research has been done recently to define appropriate standards for quantifying and monitoring AMU in livestock (12, 13). Epidemiological studies also have helped to successfully identify risk factors and drivers influencing actual AMU on cattle (14), swine (15), and poultry farms (16–18). However, the translation of research results into public policies which are able to induce change in the field remains an important challenge (19). Consequently, a series of studies were performed to analyze the cognitive and behavioral barriers to the appropriate use of antimicrobials by various stakeholders in the livestock sector. Previous studies highlighted farmers' lack of knowledge about antimicrobials and AMR (20, 21), showing that while farmers were fairly unconcerned about the risks associated with AMR, they perceived many benefits from their use (20, 22, 23). Other works have shed light on the thought processes of field actors while choosing whether or not to use antimicrobials. Previous studies have shown that such a decision emerges from a complex process in which individuals have to juggle many sociotechnical and socioeconomic elements, and that assuming actors are irrational does not provide a comprehensive framework to understand their practices and knowledge (24, 25). For example, when prescribing antimicrobials, veterinarians have to simultaneously balance animal welfare, public health and economic criteria for their clients and their own firms (26–31). These studies therefore show that we should not only analyze actors' behaviors in terms of compliance or non-compliance with standards and recommendations, but also try to understand the inner (and complex) logics framing their practices and knowledge regarding animal health and AMU. Previous studies that focused on individual behavior and decision-making processes contribute key insights highlighting the importance of empirical descriptions of stakeholders' practices and knowledge. These can be used to better tailor public policies with regard to promoting the prudent use of antimicrobials.

However, a recent article also stressed that although the intersectoral dimension of AMR has been widely acknowledged, solutions for AMR are often focused on individual behaviors, and health issues are reduced to questions of individual responsibility (32). To reconnect the individual/behavioral component of practice change with more structural elements, the analysis of AMU drivers could be deepened by exploring at least two other dimensions that have received scant attention in the literature. First, most previous studies were based on a survey design that aimed to provide an accurate picture of practices and behaviors, but which did not allow an analysis of AMU reduction as a dynamic process. In the context of changes triggered by public policies, more knowledge is needed on the temporal dimension through which actors do or do not modify the way they use antimicrobials. Previous research on the conversion of dairy cattle farmers to organic farming showed that change in AMU is a long-term, potentially reversible process (33, 34) which should be studied over time. The concept of “trajectory of change” was recently used to examine the determinants affecting the reduced use of antibiotics in swine production. Results show that actors assimilate, appropriate and implement new health practices through learning processes (35) which are progressively established over time. This theoretical framework was inspired by previous sociological and interdisciplinary research analyzing multi-level sociotechnical transition in agriculture (36). Examples involving pesticide reduction during a transition to organic farming or integrated crop production (37, 38) insist on both the dynamic and systemic aspects of change, meaning that a transition is neither the result of an individual motivation or awareness, nor of a technical innovation alone, but requires the long-term and gradual re-arrangement of several components involving social, economic, and technical aspects of farming systems. Second, literature on AMU in veterinary science has mostly focused on one stakeholder's perception and behavior, and has paid little attention to social interactions between stakeholders. Research on dairy cattle (39) and broiler chickens (40) has suggested, however, that rather than being an individual process, decision-making regarding AMU involves a complex interplay of relations between farmers, veterinarians, and farm advisors. Sociological studies on pesticide reduction and soil conservation agriculture also have highlighted the crucial role of professional networks in the success of transition pathways in farming (37). These studies in particular insist on the importance of social integration phenomena that are part of change dynamics. Indeed, farmers' practices are always anchored in social networks which form communities in which knowledge, techniques and tools are exchanged and circulate. Changes in practices are thus often paired with a change in the network which is providing farmers technical and advisory support (41).

To shed light on the transition pathways implemented in the livestock sector to reduce AMU, we conducted a qualitative study in the French free-range broiler production sector. Poultry and swine production have been identified as major drivers of antimicrobial use and subsequent development of antimicrobial resistance at a global scale (42). Surprisingly, there is a relative paucity of articles on the factors associated with AMU in poultry (43) compared to cattle and swine. In France, the free-range broiler production sector has engaged for many years in a voluntary process to reduce AMU to the minimum. This transition, which is taking place in response to quality standards and emerging societal expectations, provides a unique opportunity to simultaneously decipher the temporal and systemic dimensions associated with changes in AMU, and explore the role of close advisors, such as technicians and veterinarians, in farmers' changes in practices.

## Materials and Methods

### Selection of Participants

Three Farmer Organizations (FOs) were selected to represent both the principal production areas in France and different modes of production. We thus chose one FO from each of the two main production basins in France (in the west and southwest of the country). One FO from central France also was selected to ensure that different types of organizations were included (both cooperatives and private companies). Each FO was asked to select four farmers to represent different profiles in terms of experience (e.g., one farmer who recently began working with them and one with many years of experience) and production volumes. This target was set to meet sampling recommendations for qualitative studies regarding data saturation. It is acknowledged that the saturation point, meaning the point above which no new information appears, is generally reached after 10–12 interviews per actor category (44). In addition, interviews were conducted with technicians and veterinarians to analyze social interactions over the farmers' professional network. In each FO, two technicians were selected according to their experience in poultry breeding and their relationships with the farmers interviewed. The referent veterinarian of each FO was also interviewed. In addition, two managers (production managers in charge of AMU and FO director, respectively) were identified and interviewed in each FO.

### Data Collection

In-depth interviews were conducted face-to-face by the first author between March and April 2015. A semi-structured guide, tailored to each category of participant (farmer, technician, veterinarian, manager) was used to conduct the interviews and allow themes to emerge from the participants' narratives. The guide included open-ended questions covering the interviewee's personal and professional development, daily work (husbandry practices, relation to animals, technical, and economic performance), animal health and animal disease management, relations with other stakeholders regarding animal health (technicians, veterinarians, hatchery, feed mill, auditor/inspector, slaughter house, distributor, consumer, etc.), and use and perception of antimicrobials. To capture change over time in AMU practices, interviewees were asked to describe their personal trajectories regarding the use of antimicrobials over the past 10 years. They also were asked to identify in their life story any determining factors or triggering events related to AMU practices. We systematically put farmers' conceptions back in the frame of their work and in the context of interactions with their peers and advisors to examine interdependencies through a systemic approach.

The contents of the interviews were tape recorded, after having received each interviewee's oral agreement, and after specifying our ethical engagements of confidentiality and anonymity. The digital recordings were supplemented by written notes. Interviews lasted on average 2.15 h.

### Data Analysis

All the interviews were fully manually transcribed, and compiled with field notes. All of the transcripts were first read through to gain a sense of the data set as a whole. A thematic analysis was then performed following the methods described elsewhere (45). The first step consisted in identifying themes, concepts, and ideas that appear to be connected in some way. Text fragments were recursively grouped into categories sharing common features. In a second step, these categories then were mapped to place them in context with each other and create themes. Once all of the data were coded, similarities and differences across categories and themes were scrutinized to establish relationships and patterns. Data were progressively interpreted by putting in perspective excerpts from the data with the research question. The analysis was conducted in a circular fashion, with repetitions of forward and backward movements from text fragments, attribution of codes, and interpretation (46).

## Results

### Interviewees' Characteristics

The sample encompassed 12 farmers who represented diverse situations; for example, the poultry unit was a secondary activity for some while others were specialized in poultry farming, some produced chickens under an “antiobiotic-free” label, etc. (Table 1). The sample also included six production technicians (2 for each FO), 6 managers (2 for each FO), and four veterinarians (1 for FO “A” and FO “B,” and 2 for FO “C”).

**Table 1:** Main characteristics of the farmers interviewed.

**Interview ID**	**Age group**	**Gender**	FO^*^	**Type of buildings or huts**	**Year of installation**	**Productions on the farm**	**Description of farmer**
Farmer 1	50–55	M	A	Huts	1978	Poultry (main production) and vineyard	Took over family farm. Initial training in agriculture but not aviculture.
Farmer 2	30–35	F	A	Hut and 400 m^2^	2013	Poultry (main production) Suckler cattle Crops	Works on husband's farm (took over family farm). Converted to farming (no initial training in agriculture).
Farmer 3	50–55	F	A	Huts and 400 m^2^	1989	Poultry (main production) and crops	Took over family farm. Initial training in agriculture but not aviculture. Conversion of the wife to farming.
Farmer 4	45–50	M	A	Standard buildings + 400 m^2^	1997	Poultry (main production) and crops	Took over family farm. Initial training in agriculture but not aviculture.
Farmer 5	45–50	F	B	400 m^2^ standard buildings	1998	Poultry (main production) and crops	Works on husband's farm (took over family farm). Converted to farming (training in agriculture but not aviculture).
Farmer 6	65–70	M	B	400 m^2^	1974	Poultry	Took over family farm. Initial training in agriculture but not aviculture. Retired.
Farmer 7	45–50	F	B	400 m^2^	2002	Poultry and suckler cattle (equal parts) and crops	Works on husband's farm (took over family farm). Converted to farming (training in agriculture but not aviculture).
Farmer 8	30–35	M	B	400 m^2^	2013	Poultry	Took over family farm. Initial training in agriculture but not aviculture.
Farmer 9	40–45	M	C	200 m^2^ and 400 m^2^	1999	Poultry (secondary production), dairy cattle and crops	Does not work on the family farm. Initial training in agriculture.
Farmer 10	35–40	F	C	400 m^2^	2014	Poultry (secondary production), dairy cattle and crops	Took over family farm. Converted to farming (training in agriculture but not aviculture).
Farmer 11	60–65	M	C	400 m^2^	1977	Poultry	Took over family farm with training in agriculture and aviculture.
Farmer 12	45–50	F	C	400 m^2^	1989	Poultry and suckler cattle (equal parts) and crops	Works on husband's farm (took over family farm). Converted to farming (no initial training in agriculture).

* *FO, farmer organization*.

### Perception of Antimicrobials and Antimicrobial Resistance

General knowledge about antimicrobials and AMR was found to vary greatly among the farmers interviewed. The first difficulty when addressing the subject of antimicrobials was for farmers to properly identify the pharmaceutical class of the drugs used. Some were unsure which of the drugs that they used were actually antimicrobials. With one exception, the farmers interviewed could not provide an accurate definition of AMR; they often described it as the result of the human body becoming habituated to antimicrobials. Confusion about drug residues in meat was also observed.

“*The danger? There are residues in the meat, and the human body probably absorbs a little bit at a time. And then when you need to take an antibiotic, because everyone can get sick, the antibiotic no longer has any effect (.) Basically, the antimicrobials persist in your system, and then you cannot get cured because the human body is saturated with antibiotics.”* [Farmer 11]

Despite this confusion and inaccuracies, the main direct consequence of AMR on human health was clear for most of the farmers interviewed. They understood that AMR complicates treatments for human infections.

“*- Why do you think AMR is dangerous?*

*- We cannot be cured anymore. That's what I understand. It upsets me too.”* [Farmer 5]

Even though the majority of farmers were aware of AMR, it did not appear in the interviews as a major concern in their daily work or a threat to their health.

The farmers' main sources of information on AMR were vets and medias. FOs often offer training sessions on technical topics for their members, but none of the farmers interviewed had participated in a session dedicated to AMR. What they knew about AMR often came from unofficial talks.

“*- Has your farmer organization or the technicians provided you with some training about AMR?*

*- No, we just talked about it in passing, and sometimes on other occasions, even in relation to people. There are enough ads as it is on TV.”* [Farmer 6]

Farmers thought they had never used many antimicrobials, but acknowledged that they had used comparatively more antimicrobials in the past. Several years ago, antimicrobials may have been used even when they were not necessary from a sanitary perspective. In some cases, they were systematically administered to chicks upon arrival on the farm. Interviewees explained these misuses by the fact that free-range broiler farming in the past was not as professional as it is today, and knowledge and experience were lacking. The sanitary conditions also were different.

“*Of course there were more treatments, maybe because we had more health issues. And we were probably not as attuned to the antimicrobials question as we are today. We didn't have an all-in-all-out system and we didn't follow biosecurity measures.”* [Farmer 11]

In contrast, interviewees considered that they now are using few antimicrobials, and only when necessary. This may explain why AMU in free-range broiler farming did not seem to be an issue or a frequent conversation topic for farmers. Nevertheless, the farmers interviewed clearly perceived a trend, or a dynamic of change, in the way antimicrobials were used in poultry farming; however, this evolution was not necessarily related to a growing concern about AMR but rather to a gradual shift in their farming practices.

“*For me, antimicrobial use is not a big issue, not on my farm. Maybe other farmers have trouble. I don't know.”* [Farmer 4]

Their conviction that their AMU was low is based on the comparison with what they perceived not only of their past consumption, but also the AMU of other farmers. In particular, they assessed their AMU levels by comparing themselves with two other types of broiler production, conventional, and organic, which they used as positive and negative references, respectively. They thought they used less antimicrobials than conventional broiler farmers. They also though their AMU to be very similar to organic broiler productions because they considered their farming practices were very comparable.

“*Conventional broiler farming, it's not at all my thing. I mean, to see broilers squished together, well not squished, but enclosed in a poultry house, no. I wanted a quality product. I hesitated between organic and Label Rouge* (a French quality scheme). *I've been told that between organic and Label Rouge there was not really much difference.”* [Farmer 8]

This quote also points to the importance placed by farmers on the quality of their products. They judged they had good farming practices, ones which were sustainable and ethical for the animals, the environment and the consumer. Using as few antimicrobials as possible thus made sense for farmers, because it is part of a wider engagement to produce quality broilers. Clearly, reducing AMU has become a component of their definition of “good farming” and, therefore, of their professional identity.

“*Then, it's maybe in my philosophy; I'm not prone to taking antimicrobials. (…) I'm in favor of breeding broilers as naturally as possible.”* [Farmer 7]

Showing low AMU was also a way for farmers to convey a greener image of farming, on a topic that is usually a source of social criticism. Farmers were paying close attention to the expectations of consumers and society.

“*The request to decrease the use of antimicrobials comes from the government, but actually society is asking us to reduce it, so we have to consider this request.”* [Farmer 1]

Reducing AMU also brought economic advantages for farmers who spent less money on health issues. Some farmer organizations have started to produce broilers to market them as “raised without antibiotics.” Farmers who agreed to produce antibiotic-free broilers received extra payments for those flocks.

“*Some expenses unfortunately cannot change, I'm thinking of gas, (…) there are things on which we barely have any impact. Consumption of veterinary products, there's an impact, you chose to administer them or not. And believe me it slows lots of farmers down.” [Technician 6]*

Although financial incentives play a role in practice changes regarding AMU, some farmers also highlighted that personal beliefs and conceptions also are crucial determinants.

“*It works. It's not just something I think, it's that I know it works. (…) The wallet works. When financial compensation is not involved, there are financial penalties (…) It's a shame but hey, we are not all made the same! But I think, I am sure, that there are better results with people who are really convinced than with those who become convinced because they get some money. Money is a means but it is not the best.” [Farmer 9]*

### Technical Factors Involved in AMU Reduction

Analysis of the interviews also sheds light on the way farmers managed to reduce their AMU. First of all, farmers associated better health and reduced AMU with improvements in chicks and feed quality.

“*Because now, you have to admit it, hatcheries are delivering perfect goods. Whereas before, what could you do when you received a flock of sick chicks? It was neither the farmer's fault nor the feed's fault.” [Farmer 6]*

Farmers also explained that AMU reduction was made possible by the adoption of new practices such as the acidification of water. This is propelled by discussions with professionals from the poultry sector (sales agents, technicians or vets), but also by recommendations from other farmers or after a farmer had experimented with it on other livestock. One farmer explained he first learnt how to use the acidifier on his duck production unit, and then extended it to all of his poultry production units as he judged that he used fewer antimicrobials since adopting this technique.

Probiotics and herbal drugs were other examples of products recently adopted by farmers. Most herbal drugs are currently classified as a dietary supplement in France. That implies that farmers have free access to these products, and can quickly react to a health issue. These products were recommended by some FOs to be used as a prophylaxis, but technicians also used herbal drugs as an alternative to antimicrobials to manage some health issues. Interviewees highlighted that the adoption of new practices such as herbal drugs relied on the ability of technicians and veterinarians to show the proof of their efficacy.

“*It's true that once they have seen that herbal drugs work, they tend to continue with herbal* drugs, rather than go back to chemical drugs.” [Technician 4]

Farmers carried out multiple experiments, mainly with alternative medicines. Experiments can be initiated on the request of the FOs, which recruit farmers to carry them out. One farmer also explained how he developed his own experiments on the management of water quality, without any collaboration with the FO's technical staff. After having successfully decreased the occurrence of digestive disorders in chickens (and in turn reduced antimicrobial use), he showed his neighbors how to lower the pH of water. This example shows how learning progressively passes from one farmer to another.

Farmers progressively have been adopting more preventive approaches to manage their farms. Most of the practices mentioned by farmers as levers for reducing AMU were actually not innovative by themselves (cleaning and disinfection, prophylaxis, etc.). What farmers emphasized is that the changes that occurred did not involve the adoption of new tools or practices as much as improving how they implemented some existing practices. In a nutshell, it was less what they did than how they did it. They mentioned, for instance, the improvement in cleaning and disinfection operations, and in the respect of a strict downtime between two flocks.

“*Now we have a more preventive approach. I think we've been taught to decontaminate water pipes.” [Farmer 11]*

Technical advisors also highlighted the critical role of biosecurity as a lever for AMU reduction. The main difficulty relies in their effective implementation of good practices in the field, and the maintenance of farmers' compliance over time. In this regard, the recent epidemics of highly pathogenic avian influenza in France have brought to the fore the crucial role of well-known biosecurity measures, and offered technical advisors opportunities to insist on biosecurity compliance. These episodes were used by technical advisors as an opportunity to prompt change in biosecurity practices.

“*We've been fighting for biosecurity every day, ever since 2006 (.) and now we are finally succeeding. Basically, the regulations and the current situation in France are a huge help (…), it has been an opportunity clearly answer the question: what is biosecurity?(…) When someone says, “Why do I have to change, I don't understand, my chickens, they go outside, you are full of nonsense.” (.), now I can respond: “You are doing biosecurity today for influenza”, but it is actually for all diseases.” [Manager 3]*

Farmers also stated that they have progressively modified their reaction when facing a syndrome, including a mortality episode, in the flock. Farmers described how, instead of treating with antimicrobials as soon as the problem starts, they were now waiting to see how it evolves. Sometimes clinical signs ceased quickly by themselves; waiting and watching the flock closely enabled farmers to avoid AMU. This is actually an example of collective learning within the professional network, because technicians and veterinarians also had to modify the way they react when a farmer calls to notify mortality.

“*I wouldn't have agreed to wait and see without treating because I was young, inexperienced. I didn't know it was useless. But today when some of my animals are coughing, I don't rush to the vets. Because I know it's going to stop in a few days.” [Farmer 7*]

### Role of Interpersonal Relationships in AMU Reduction

All of the stakeholders mentioned that the technical support provided to help farmers solve health issues in their flocks would not be effective without well-established confidence relationships between farmers, technicians, and veterinarians. According to actors, this confidence relationship was also absolutely necessary for farmers to accept the risk of waiting before treating in situations with a case of mortality.

Interviewees highlighted that trusting relationships are formed progressively, and that the degree to which actors invested time in these relationships was critical.

“*Concretely, we have to pass a lot of time with them [the technicians], they have to trust us, and we have to talk to them about lots of things other than poultry. You have to create a relationship (.) You can talk about their work, or about plenty of other things, and then they gain confidence, and they listen. But it takes time. It's a relationship.” [Veterinary 2]*

Actors highlighted that three main mutual commitments were necessary for establishing this relationship of confidence. First, veterinarians, technicians and farmers shared a responsibility because any inappropriate practice could damage the whole production organization. Second, they had to remain humble and question themselves when facing a problem. Third, actors also had to stick together and help one another. Farmers were not alone; technicians and veterinarians committed themselves to being reachable on weekends and bank holidays.

“*I tell farmers, ‘When you have sick animals, call your technicians so we can decide what to do together, what level of risk we should take’. It's true that Friday always is a challenge, and farmers say: ‘Wait, how are we going to spend the weekend!’. (…) I give them my number, so we take stock together. (…) We've got to make farmers feel that we are with them, we have their backs. If you leave them to struggle with their own fears, they are going to take the easy way (…). So you have to stay by their side, etc. they shouldn't be alone. When a farmer has done this with you once, (…) he no longer needs this positive feedback precisely because he has made his own paradigm. He says to himself, ‘If it is possible, we can do it, I have already succeeded once.”’ [Manager 3]*

The trusting relationships established by farmers, veterinarians and technicians have even made it possible to share the decision-making process regarding the use of antimicrobials. To a certain degree, the choice of a given intervention, while theoretically the sole domain of veterinarians according to the French Public Health Code, integrates the expertise of both the farmer and the technician who first intervenes when there is a problem on a farm.

“*When animals fall ill, the technician is called. He goes there and does an autopsy. If he identifies what the problem is, he manages things himself. If he's not sure, he usually calls us, and then we discuss it. In some ways, he is like our eyes. We do a lot over the telephone because the technicians have already done the legwork. So you definitely must have a relationship of trust. One has to be absolutely sure that they're not going to tell us a load of rubbish. But normally, it's true that legally, it's not supposed to happen like that.” [Veterinary 2]*

Gradually, the close relationship formed between a technician and a farmer allows personalized adjustments. The technician draws from his or her personal knowledge of the farmer to encourage good practices, using whatever argument s/he deems to be the most persuasive under the circumstances [economic (cost of treatment), fear of being audited, or benchmarking with peers].

### Role of Collective Actions in AMU Reduction

Our results highlight the key role of downstream operators (distributors and slaughterers) in the beginning of AMU transition pathways. Regular audits prompted by the request of downstream operators do not simply verify conformity with specification standards. They are used as an opportunity to initiate change in an FO's practices.

“*It was really with this audit that we said OK, we have to do something because next year he [the auditor] will ask us to do it anyway, so we might as well go ahead and develop something for the technicians to make it more efficient.” [Manager 1]*

Downstream operators clearly asked FOs to put in place monitoring tools that allowed AMU to be evaluated in a quantitative manner. The development and adoption of such tools by the FOs had a positive impact, making it possible to become aware—based on factual data—of AMU practices on farms. Once AMU was closely monitored, FOs started developing “antibiotic-free” labeling strategies for positive market differentiation. Farmers and managers emphasized that the use of these tools was even more effective than the financial bonus associated with “antibiotic-free” production to reduce AMU. The tools made it possible to quantify and visualize use, enabling farmers to compare their performance with that of others, and a dynamic of emulating good practices was established and further rewarded by labeling.

“*Antibiotic-free chickens was something that definitely helped us reduce antibiotic use. It is clear, because I think that we all ended up being very invested in this, the technicians, vets, farmers, and the group of farmers who adhered. Sure, the bonus had an effect, which surely also made it possible to do a certain number of things. But I think that gradually it even became like a kind of challenge to say: We are getting there so we should no longer use antibiotics.” [Manager 2]*

In addition to quantitative tools, downstream operators also have the power to ask for qualitative changes in practices, with reduction of the use of critical antibiotics.

“*We made a progress plan together with this client, who is a client with whom we have a really close partnership. Once we had the plan, the client wanted us to move forward on it. He said that he didn't want, and wasn't expecting, zero-treatment chickens, but rather a positive dynamic, meaning better management of antibiotics.” [Manager 6]*

To reduce AMU, FOs have progressively developed various mechanisms which aim to engage farmers in a transition pathway that is shared at a collective level. To encourage farmers to spontaneously join collective actions to reduce AMU, FOs have been mobilizing levers such as the shared identity of farmers, their pride in being a farmer and a sense of belonging to a community. One FO clearly stated that the objectives of its strategy were to create a new social standard that farmers would want to comply with by joining a specific action. This action was based on the establishment of a multicriteria performance score which aggregated various indicators and could penalize treatments of increasing severity (with, for example, an antibiotic treatment associated with a higher penalty than a treatment based on herbal medicine). The expected result, from the FO's point of view, was that farmers would voluntary enroll in the process, and their “culture” would progressively be modified through the embrace of this new approach. Ultimately, farmers would change their practices simply by embracing a collective challenge. Rather than pushing them to change their practices, the FO was asking them to change their “state of mind.”

“*Once again, the point of this project is to get the employees and the members on board in a process whose ultimate goal is to improve performance and reduce antibiotic use because we will lower antibiotic use through everything in the project. I mean there is no one factor that will alone reduce use, there is the state of mind (…). By being part of the process, farmers are going to start asking themselves the question. I am hoping that by creating the dynamic, farmers will say to themselves: ‘Damn, I have to use an antibiotic, that bothers me.’ Whereas before, the reaction was:' The birds are sick, let's get out the antibiotics', you see what I mean?” [Manager 4]*

The goal was to induce farmers to enroll in a comprehensive approach that had multiple objectives. Most of the time, reducing antimicrobials was not the sole focus, but rather one component in a combination of changes that aimed to increase overall performance (which was not only assessed in terms of productivity or profitability, but also of complying with certain values and professional identity). Reduction in antimicrobial use as it was addressed by the FOs was therefore complex and not an objective in itself, but served in fact several purposes. With the exception of one FO, most of the tools mobilized were not developed specifically to reduce AMU. Rather, they were items designed for another strategic function that were later applied to reduce AMU (e.g., a smartphone application first developed to manage internal controls and salmonella tests). As a result, the management of actions related to AMU is often assigned to several different actors for whom AMU is an added duty on the fringe of their main mission. This reflects the fact that the FOs do not yet have a clear vision of the economic impact of changes in AMU, and have therefore decided to divide tasks between existing staff rather than creating a position dedicated only to AMU reduction.

The three FOs finally reached the conclusion that reducing AMU was a “management” issue that needed to be spearheaded and coordinated at the FO level rather than left to the initiative of individual farmers. They highlighted the importance of a collective strategy piloted by a lead actor who, convinced of the importance of the subject and with a vision for his or her company, decides to set up actions such as appointing an AMU coordinator and putting in place an elaborate framework for a transition pathway toward AMU reduction.

“*In the end, not that many people are orchestrating this. (…) The main obstacle, as we mentioned earlier, is linked to systems—both the education system and the industrial operating system—which compartmentalize. Therefore, what is perhaps the most exhausting part is to explain to people that it is necessary to decompartmentalize, that it is necessary to train to become like a pilot (…). We need to bridge the gap between advice and practice. (…) Ultimately, if we want to define actions and measure them, a coordinator must be someone who is not busy with something else, we need to afford the luxury of having someone with health skills who gets paid to do nothing but that.” [Manager 2]*

## Discussion

Debates about appropriate methodologies for studying public health problems have long been polarized by the opposition made between quantitative and qualitative approaches (47). Epidemiological approaches have been widely used to hierarchize the respective role of risk factors and analyze the causal link between farming practices and AMU on farms. On the other hand, sociological research and qualitative methodologies aim to gain an inside view of the context and intentions underlying professional practices and knowledge, and to document interactions and power relations between the various stakeholders. This article rather leans on this second body of work and uses it to discuss interdisciplinary approaches which have been developed these last years in the field of animal health, and AMU in particular.

However, we should first of all expose some limitations of the study. The sampling strategy and the theoretical models we used certainly limit results generalization to very specific production sectors. Although the selection criteria used in the sampling and the data saturation which occurred in the analysis allowed us to document a wide range of practices regarding antimicrobials use in free-range broilers in France, our results may not be applicable to all animal production systems. Indeed, since organizational characteristics of the value-chain and of the labor division within FOs have been proved to be a decisive factor of AMU decision-making process, our conclusions could be only applicable to animal production systems sharing similar characteristics, such as the pig and poultry industry which are known for more integrated socio-economic structures. We also acknowledge that some AMU practices may have changed since the time of the study. However, it is likely that those changes are limited as (i) interviews suggested that major changes in AMU (e.g., reduction of metaphylactic use) had already occurred before that time, and (ii) AMU was already low at the time of the study (16). Moreover, as the objective of this article is not to provide a quantitative assessment of AMU but to analyze the drivers of AMU practices and knowledge (stakeholders' interactions, labor division, organizational characteristic of the FOs, etc.) which shape long-term farmers' trajectories, our conclusions should thus remain valid for other studies. Consequently, the present work, based on a case study of the free-range broiler sector in France, brings new insights on the transition pathways toward an optimized and prudent use of antimicrobials.

First, farmers identified that technical improvements are a key success for reduced AMU, in particular the quality of inputs (feed, chicks), use of alternative medicines and biosecurity. These results are in line with previous studies. The quality of feed and chicks has been found to be decisive for chicks' health (48) and associated with variations in AMU in broiler production (18). In addition to these well-known technical factors, farmers also emphasized the role of alternatives to antimicrobials. The efficiency of alternatives such as herbal drugs has not yet been demonstrated, and experimental proof is obviously lacking. However, a recent epidemiological study carried out on the same study population (16) showed that the use of herbal drugs was associated with a decreased probability of AMU in the field. As farmers and health advisors are increasingly interested in the use of alternative prevention strategies (including vaccines, prebiotics, probiotics, and herbal drugs), further studies are needed to assess their effect in relation with AMU reduction. Results highlight that the adoption of these new technical tools (acidification of water, use of herbal drugs) is a progressive process, in which on-farm experiments have a key role. The implementation of such experiments depends on farmers' motivations, the appeal of novelty, and the advice farmers may receive from technical advisors (technicians, veterinarians). Once actors perceive that a form of “proof” has been established, the practice can spread through informal exchanges that occur between farmers, or be incorporated into a formal transfer program such as training activities set up by FOs. The positive impact of joint learning has already been studied in intervention studies in the context of Danish Stable School (49). All of the stakeholders (farmers, technicians, veterinarians, managers) also identified biosecurity as a crucial lever for managing AMU on farms. In France, recent episodes of highly pathogenic avian influenza that seriously impacted the poultry sector (50) obviously put the spotlight back on this “well-known” tool for disease control. The relationship between biosecurity measures and AMU on farms is, however, complex; thus far it has mainly been investigated in the pig sector. Results from a study carried out in German farrow-to-finish farms found that the level of biosecurity of herds was associated with the amount of antimicrobials used (51). Similar observations were made in a multi-site cross-sectional study conducted on pig herds from Belgium, France, Germany and Sweden (52). In contrast, no clear association was found between biosecurity and antimicrobial consumption in the context of Danish pig farming, which presents generally high biosecurity and many years of official restrictions regarding antimicrobial use (53).

Second, results highlighted gaps in knowledge regarding AMU and AMR. As a previous study demonstrated that the level of farmers' knowledge was significantly and inversely related to AMU at the farm level, whatever the species considered, efforts need to be pursued to heavily target knowledge of AMU and AMR in communications with veterinarians and through educational campaigns for farmers (54, 55). Regarding farmers' perceptions, one of the main drivers identified in the present study for antimicrobial reduction was alignment with farmer professional identity and sense of good farming. Implementing a new practice that contradicts farming identity complicates and even prevents its adoption by farmers who happen to be very sensitive to what their peers are doing, and often compare their practices (56). In a previous work examining the social factors that influence the length of the antimicrobial treatment administered to dairy cows for mastitis (54), the authors showed that giving an antimicrobial treatment over an extended period despite the injunction to reduce AMU enabled farmers to comply with the social norm of “being a good farmer” that was conveyed by their peers, vets and advisors. Our study suggests something slightly different. Indeed, even though professional networks are a key component of farmers' decision-making process, there was clearly no identity break associated with decreasing AMU. On the contrary, French traditional free-range broiler farmers have built a conception of their work that values AMU reduction. It was in particular expressed through the fact that they attached importance to consumers' opinion and were eager to prove that they produce quality products. All in all, farmers' knowledge and practices, among which their attitudes toward AMU, are associated with farming subjectivities which are equipping farmers with a certain sense of good farming, that is to say attributing positive values to a certain type of farming (56). Of course, these subjectivities are also the product of larger social structures which in our case mostly relate to the professional network and the FO, but in some cases can be more directly connected to national agricultural policies (57). For instance, farmers' identity has been used as a driver for change by one of the FO analyzed, which clearly stated an objective of changing the “culture” or “state-of-mind” of farmers to accompany them toward a reduction of AMU.

Third, our results showed that farmers' embeddedness in collective organizations is decisive for farmers to accept and change their practices. Analyzing AMU reduction should not rely on a conception of change made from an individualistic or behavioral point of view, but should rather try to understand how the structure of the sociotechnical and socioeconomic networks in which farmers are embedded favors change or not (or what kind of change it favors). Results of our study showed that the FOs act like a professional network for farmers, providing technical advice, inputs supply and products commercialization. Farmers and technicians know each other, and technicians know which incentive can motivate farmers to implement some practices, and thus deliver advice that is personally adjusted to farmers (40). The confidence relationship between farmers and their advisors underpins the moral support that technicians and veterinarians provide to farmers. Palmer, Sully et al. (58) have for example shown the importance of a trusting relationship for the implementation of biosecurity measures in livestock farms. In our case, this moral support provided by farm advisors was decisive to help farmers considering making any change and accepting to take a risk, such as mortality. As showed by Fortané et al. (35), farmers have to operate a cognitive change by modifying their perception of risk while learning to wait before treating in case of mortality. The farmers we met learnt that in some cases treating the flock with antimicrobials may enable puny broilers to be saved, but the latter will always have a lower weight than the other birds and ultimately penalize the flock. Accepting a certain level of mortality during a short period of time in the flock is a way to accept natural sorting among birds. This change in the attitude toward risks and antimicrobial treatment was of course related to a change of their conception of what a good farmer is to someone who does not necessarily have to act as quickly as possible to stop mortality and disease in his or her flock. Lamine et al. (37) observed a similar change in farmers who had converted to integrated crop protection, and who changed the hierarchy of the accepted risks. They delayed their seeding in order to avoid diseases and decrease crops treatments, but on the other hand, they had to accept the higher risk of rain. By accepting a risk which they did not accept before conversion, they also changed their conception of what “beautiful wheat” is. It is also noteworthy that economic incentives did not appear in our study as a major driver for change in AMU compared to technical and cognitive factors, with the exception of a financial bonus for breeding antimicrobial-free broilers. This finding is in line with previous work that showed that financial incentives and penalties are inefficient if farmers do not intend to change (59).

All of these elements shed lights on different aspects of agricultural transitions and practice changes (36). First, transitions are always systemic, which means that it is not enough to simply withdraw a sole element (for example, antimicrobials). This withdrawal implies a global (though not necessarily radical) rethinking of the system. In the present study, farmers experimented with alternative medicines of different natures, they also implemented new biosecurity measures and tried different strategies, mostly preventative, to manage animal health. These systemic elements are not just technical or economic. It is important not to forget the social, cultural, and cognitive components of farming systems since transitions also encompass phenomena such as professional relationships, a sense of good farming and perception of risks. Second, transitions are dynamic, which means they have to be understood as a mid or long-term process and often from an incremental perspective. They cannot just be related to motivations or trigger events inducing radical changes, as even though these aspects matter in many cases (60), including in AMU reduction (35), they should not necessarily be considered as a starting point of a trajectory of change. Analysis of verbatims showed that for the free-range poultry farmers we interviewed, the decision to reduce AMU was not triggered by a specific event, but was part of a broader dynamic of change that was fostering this transition toward more sustainable farming. Reducing AMU was part of this change, but was not the alpha and omega of it and more importantly, these changes occurred progressively. Previous works on transitional pathways in organic crop farming have shown that a farmer's experience is essentially built and adjusted through experimentation related to the introduction of a new technique (61). Our results show that adoption of new preventive practices (such as acidification of water, or use of alternatives to antimicrobials) was a progressive process in which farmers' experiences and relationship with risk were gradually recomposed over time. The farmers did not mention brutal changes, but a change in continuity (62), even for technical improvements which are often made on an incremental basis. This finding supports those from a previous study, suggesting that a change of practices related to AMU on pig farms was shaped over a relatively long period of time (35).

In addition to technical factors, our results also highlight the importance of time in the establishment of trusting relationships between actors, which are a crucial prerequisite for farmers' acceptance of the risks associated with AMU reduction. This is linked to the point that the transition pathways we observed in our case-study did not involve a withdrawal from the sociotechnical networks in which the farmers were embedded. On the contrary, these networks (materialized by the professional relationships with vets and advisors, as well as the technical and economic support of the FO) actually enabled changes and transitions in farming practices. This result is quite distinct from other cases described in the literature, for example for transitions toward soil conservation agriculture or pesticide reduction, where the strong ties of the traditional sociotechnical network had to be broken in favor of weaker ties that could then be strengthened with more alternative networks (37, 41). This could be interpreted as a consequence of the specificity of our fieldwork. We studied three relatively small FOs in a quality sector where the relationships between farmers, technicians and veterinarians are close. Furthermore, the farmers we interviewed had strong confidence in their FO to help them work in a way that fulfilled their professional identity, and so they trusted the strategy offered by their advisors to reduce antimicrobials. Perhaps more importantly, we studied a quality label sector that was already providing forms and senses of sustainability to farmers, who did not feel that reducing AMU was a massive change in their way of farming and of being (good) farmers, so that the transitions in which they engaged felt like a “natural” continuity in their career and the trajectory of their businesses. All in all, our study sheds lights on the diversity of agricultural transitions.

## Data Availability Statement

The datasets presented in this article are not readily available because sharing publicly full excerpts would compromise the agreement to which the participants consented. Requests to access the datasets should be directed to MP, mathilde.paul@envt.fr.

## Ethics Statement

Ethical review and approval was not required for the study on human participants in accordance with the local legislation and institutional requirements. The patients/participants provided their written informed consent to participate in this study. Written informed consent was obtained from the individual(s) for the publication of any potentially identifiable images or data included in this article.

## Author Contributions

All authors contributed conception and design of the study. CA collected the data, performed the thematic analysis, and wrote the first draft of the manuscript. NF and MP wrote sections of the manuscript. All authors contributed to manuscript revision, read, and approved the submitted version.

## Conflict of Interest

The authors declare that the research was conducted in the absence of any commercial or financial relationships that could be construed as a potential conflict of interest.
